# Combined Mohs Micrographic Surgery in a Collision Tumor

**DOI:** 10.7759/cureus.26747

**Published:** 2022-07-11

**Authors:** Rafael A Montealegre, Juan C Barrera

**Affiliations:** 1 Dermatology, Instituto Nacional de Cancerología, Bogota, COL

**Keywords:** external ear, frozen section, paraffin embedding, lentigo maligna melanoma, basal cell neoplasms, mohs surgery

## Abstract

We present the case of a 73-year-old male patient with a collision tumor in the right ear, consisting of a basal cell carcinoma and melanoma in situ. He was managed with Mohs micrographic surgery combining paraffin-embedded sections and frozen sections. Multiple surgical stages were required to obtain tumor-free margins. The surgical defect was reconstructed by plastic surgery, achieving the preservation of the ear. The technique of combining two processing sections is useful in the treatment of the collision of basal cell carcinoma with malignant melanoma.

## Introduction

Skin cancer is the most common malignant neoplasm in humans. The three most common tumors are basal cell carcinoma, squamous cell carcinoma, and melanoma [1]. There are several treatments described for these tumors, including Mohs micrographic surgery [2,3].

Mohs micrographic surgery is a surgical and histopathological technique that allows a 100% evaluation of the peripheral and deep margins. It is useful in skin tumors on the head and neck, in particular, to achieve narrow resection margins and spare healthy tissue [4].

It is especially important to have a deep knowledge of the anatomy of the site where the Mohs micrographic surgery is going to be performed, as this has different implications for both the time of resection and the time of processing of the surgical specimen [5].

## Case presentation

**Figure 1:**
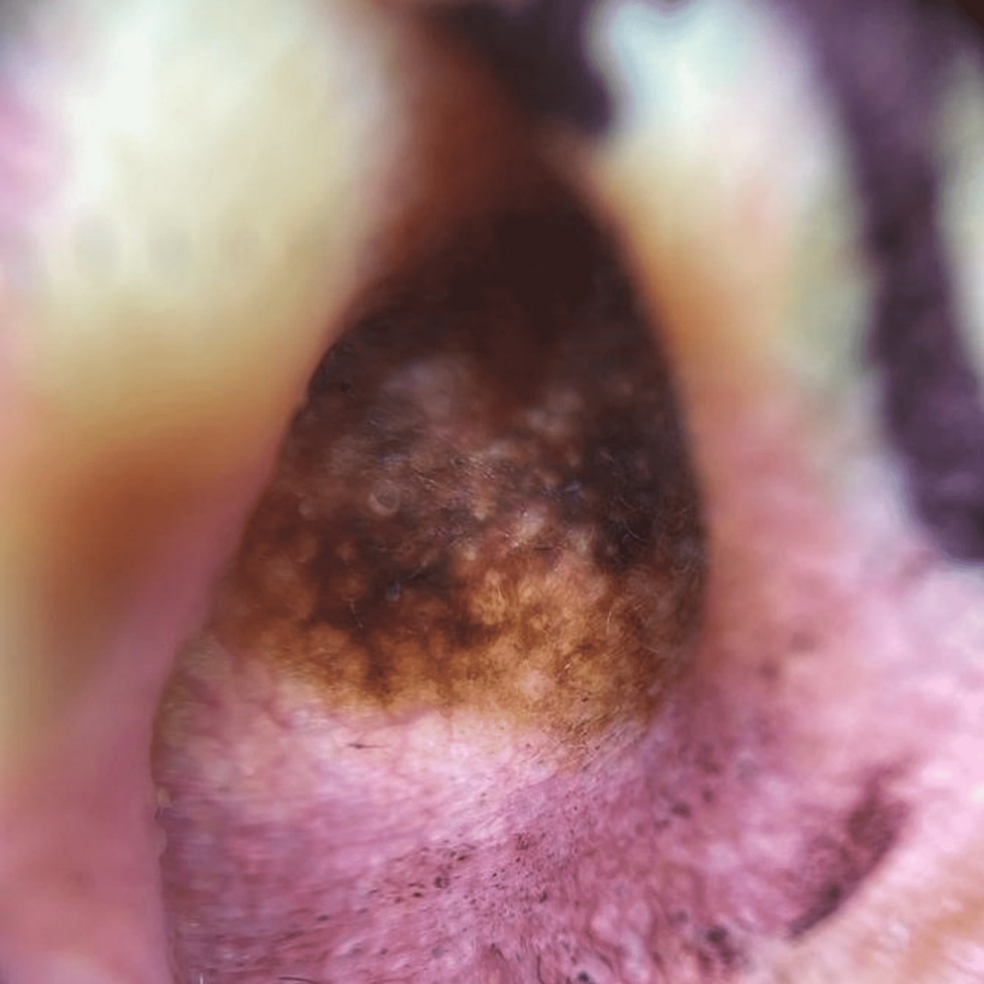
Dermatoscopy of lintigo maligna located in the concha cymba, showing pseudonetwork with asymmetric follicular openings and rhomboid structures.

**Figure 2:**
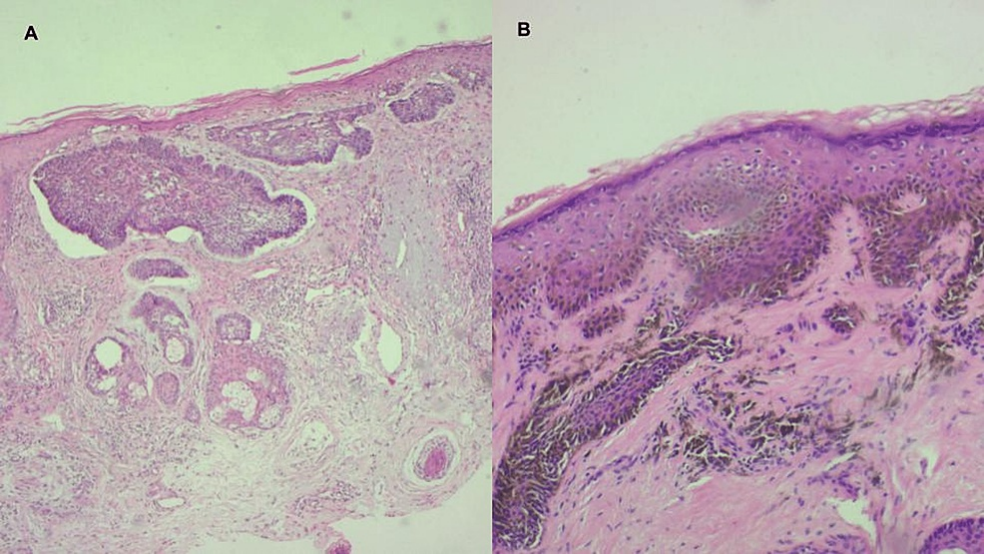
Histological findings. (A) Diagnostic biopsy of nodular and superficial basal cell carcinoma in the concha cava. (B) Diagnostic biopsy of lentigo maligna in the concha cymba.

A 73-year-old male patient, with no relevant medical history, was referred to our institution for an infiltrative basal cell carcinoma in the cavum conchae on the right ear. In addition, during the consultation, we found a pigmented lesion on the ipsilateral cymba conchae. We performed dermatoscopy and found a pigment pseudonetwork with asymmetric follicular openings and rhomboid structures (Figure 1). After this finding, a biopsy was taken, which revealed lentigo maligna (Figure 2).

**Figure 3:**
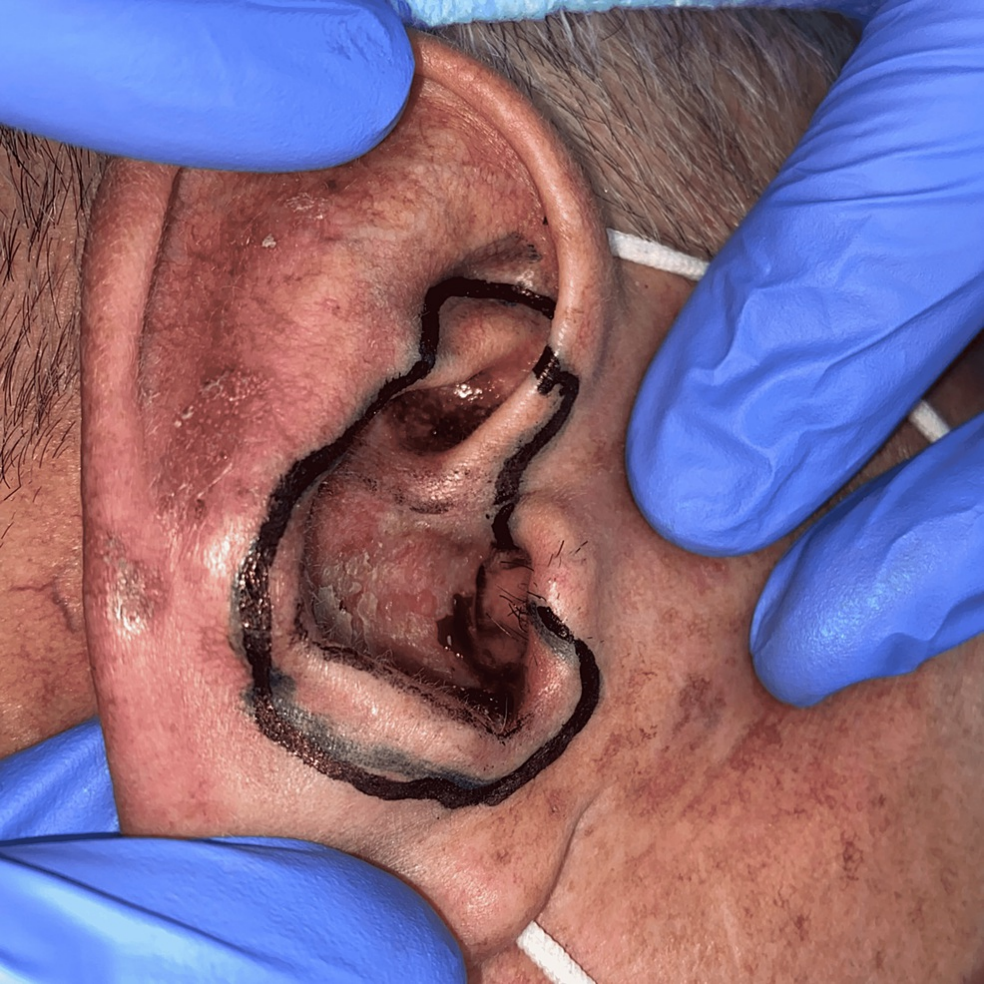
Demarcation of infiltrative basal cell carcinoma in the concha cava and lentigo maligna in the concha cymba.

**Figure 4:**
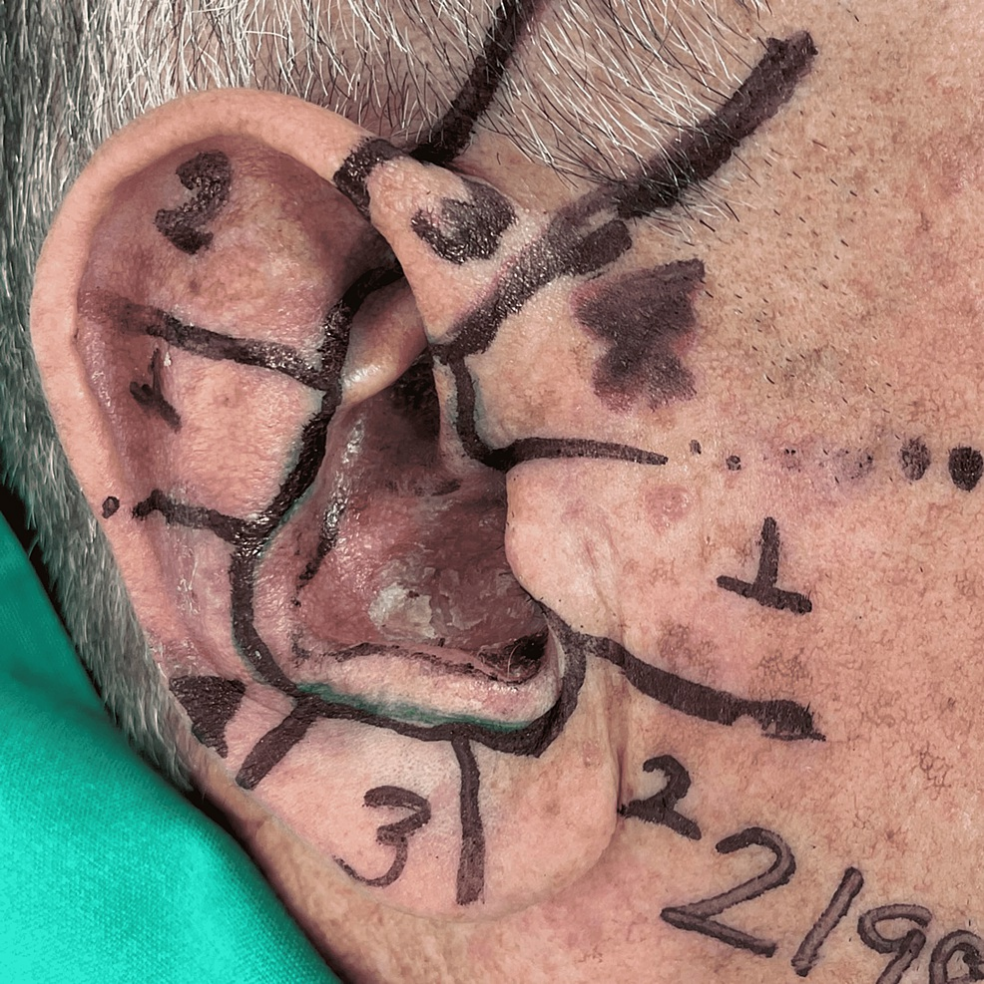
Surgical planning shows that the margins of the lentigo maligna (upper) are delimited into four fragments. The margins of the infiltrative basal cell carcinoma (lower) are delimited into four fragments.

We decided to perform Mohs micrographic surgery for collision tumors (basal cell carcinoma and lentigo maligna). During the same surgical time, we used frozen sections for basal cell carcinoma and paraffin-embedded sections for lentigo maligna (Figures 3, 4). All pathology slides were reviewed by the Mohs surgeon and later by the Pathology Department for the purpose of quality control.

**Figure 5:**
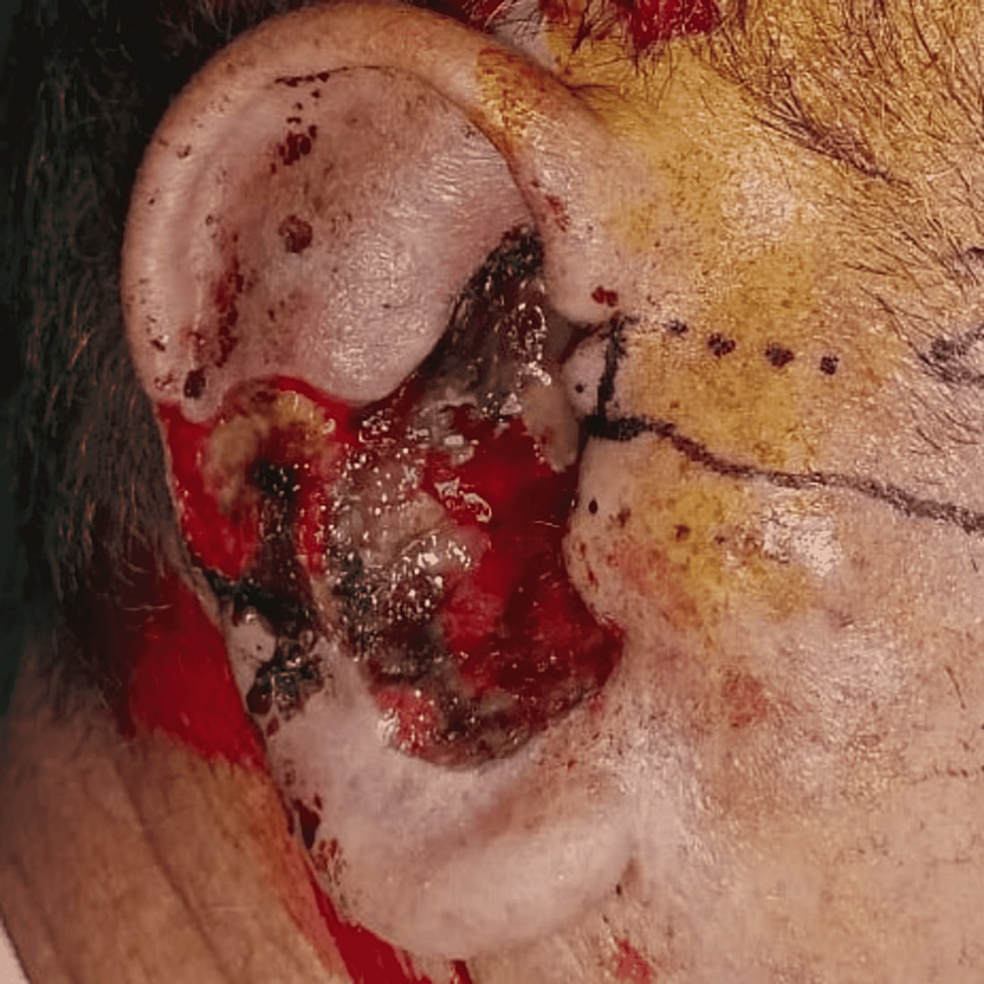
Surgical defect of Mohs micrographic surgery after four stages in basal cell carcinoma and one stage in lentigo maligna.

**Figure 6:**
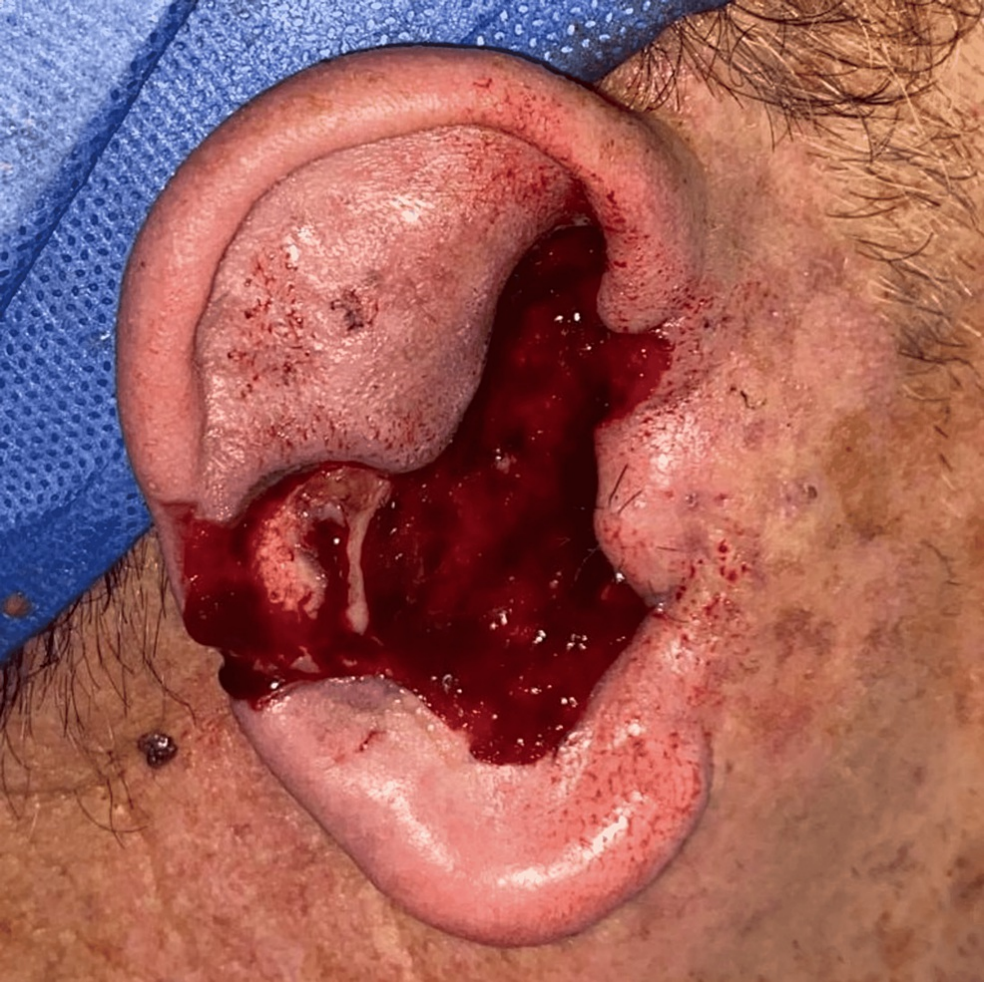
Surgical defect of Mohs micrographic surgery after four stages in basal cell carcinoma and two stages in lentigo maligna.

**Figure 7:**
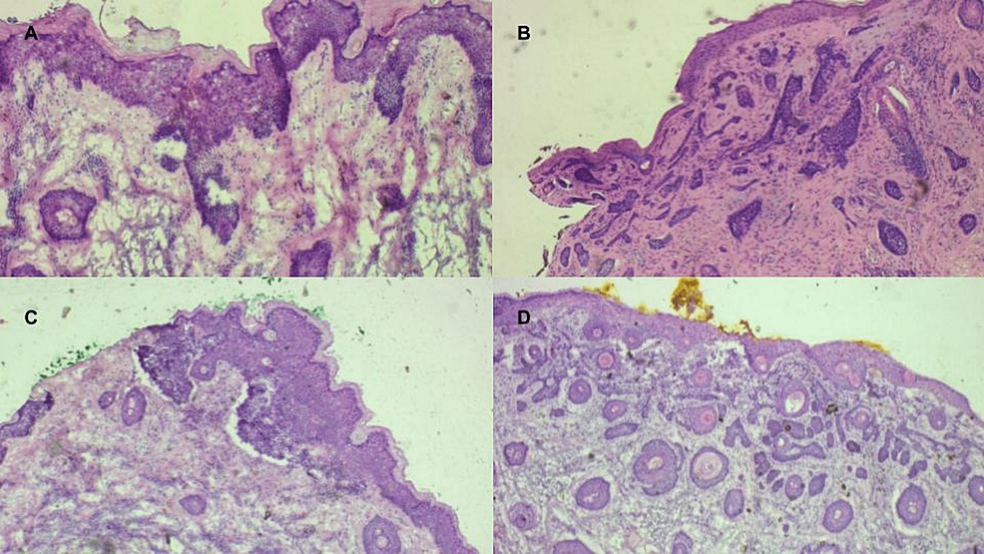
(A) First stage result of the concha cavum, showing the presence of superficial basal cell carcinoma in fragment 4. (B) Second stage result of the concha cavum, showing the presence of infiltrative basal cell carcinoma in fragment 1. (C) Third stage result of concha cavum, showing the presence of superficial basal cell carcinoma in fragment 1. (D) First stage result of concha cymba, showing the presence of superficial, nodular, and infiltrative basal cell carcinoma in fragment 4.

**Figure 8:**
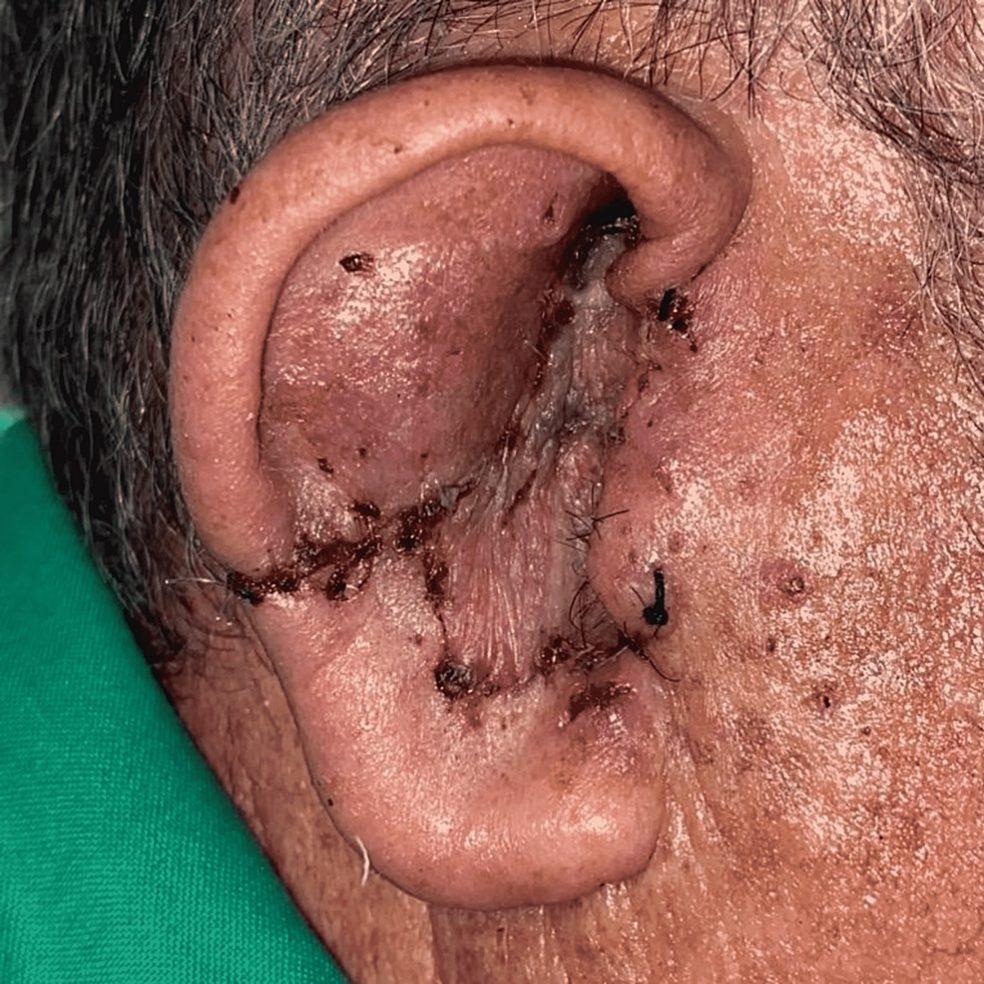
Reconstruction result showing wedge resection of the helix cartilage with primary closure using a total skin graft for the concha defect.

We performed four stages on the basal cell carcinoma fragment processed by frozen sections: two stages due to superficial pattern involvement, one stage due to infiltrative pattern involvement, and the last stage presenting margins free of tumor involvement (Figure 5). The fragment of the lentigo maligna processed with paraffin-embedded sections did not show compromise by lentigo maligna in its section margins during the first stage. However, it was compromised by superficial and infiltrative basal cell carcinoma, which is why we performed a second stage by using frozen sections. This second stage with frozen sections presented margins free of tumor involvement (Figures 6, 7). Reconstruction was performed by the plastic surgery unit for which they made a wedge resection of the helix cartilage with primary closure and used a total skin graft for the concha defect (Figure 8).

## Discussion

Collision skin tumors are a common finding in both benign and malignant tumors. However, the collision of basal cell carcinoma and melanoma is infrequent and found in 1.4 per 100,000 basal cell carcinomas [6]. Regarding the terminology to be used in the context of basal cell carcinoma and melanoma, Satter et al. proposed the following four concepts: Collision tumors are two independent neoplasms that occur very close to each other, maintaining defined limits. Combined tumors are when there is a neoplasm composed of two distinct but intertwined populations of malignant cells. Colonized tumor, when melanoma in situ, permeates an underlying basal cell carcinoma, with the proviso that atypical melanocytes must remain within the boundaries of the basal cell. Finally, biphenotypic tumor arises from a common stem cell precursor undergoing divergent differentiation, with the two tumor populations exhibiting overlapping immunohistochemical and molecular properties [7].

The ears have a special cosmetic and functional relevance. Skin tumors in the external ear appear to be more frequent in male patients and tend to have a worse prognosis compared to other locations on the body. The most prevalent neoplasms in this location are squamous cell carcinoma and basal cell carcinoma followed by melanoma [8]. In addition, the ear has a characteristic complex topography that makes it very difficult to perform surgical and frozen section procedures. This makes it more difficult to obtain tumor-free margins and preserve healthy tissue at the same time [5].

Currently, the most widely used processing technique in Mohs micrographic surgery is frozen sections. However, the use of paraffin-embedded sections has been described for lentigo maligna, also called "slow Mohs." in order to reduce false-positives, as there are no paraffin artifacts that could be confused with tumor melanocytes [9]. On the other hand, standard recommendations cannot be applied to collision tumors, and treatments must be individualized based on the type of tumor (which one has the worst prognosis?), location, and patient status [10].

As additional considerations for this case presented, frozen sections were not performed with immunohistochemistry since we have not yet built a significant experience for its use. Surgical management was preferred over topical management due to the low response of basal cell carcinoma to this type of treatment. The reconstruction was performed by the plastic surgery unit due to regulations in the institution; however, we consider that it can also be performed by the same Mohs surgeon.

## Conclusions

In the case presented, we showed a collision tumor composed of basal cell carcinoma with an infiltrative pattern and a lentigo maligna, located in an area of difficult surgical approach such as the auricular concha. In addition, we carry out the treatment with Mohs micrographic surgery combining paraffin-embedded sections and frozen sections, which spared the patient’s healthy tissue, and tumor-free section margins were obtained.

We agree with other authors that the management of this type of collision tumor must be individualized. Mohs micrographic surgery is a technique that can be individualized as per the needs of each patient. The combination of paraffin and frozen sections is a very good tool for the treatment of basal cell carcinoma and melanoma in collision tumors.
